# COVID-19 and Global Health Security: Overview of the Global Health Security Alliance, COVID-19 Response, African Countries’ Approaches, and Ethics

**DOI:** 10.1017/dmp.2020.360

**Published:** 2020-10-02

**Authors:** Belete Yimer, Wassachew Ashebir, Awraris Wolde, Muluken Teshome

**Affiliations:** Department of Public Health, Debre Markos University, Debre Markos, Ethiopia

**Keywords:** Africa, COVID-19, ethics, global health security, pandemics, public health response, solidarity

## Abstract

Public health emergencies can arise from a wide range of causes, one of which includes outbreaks of contagion. The world has continued to be threatened by various infectious outbreaks of different types that have global consequences. While all pandemics are unique in their level of transmission and breadth of impact, the 2019 coronavirus disease (COVID-19) pandemic is the deepest global crisis of the 21st century, which has affected nearly every country globally. Yet, going forward, there will be a continued need for global health security resources to protect people around the world against increasing infectious disease outbreaks frequency and intensity. Pandemic response policies and processes all need to be trusted for effective and ethical pandemic response. As the world can learn during the past few years about frequent infectious disease outbreaks, (these) diseases respect no borders, and, therefore, our spirit of solidarity must respect no borders in our efforts to stop the ongoing COVID-19 pandemic and be better prepared to respond effectively to a health crisis in the future.

## INTRODUCTION

Public health emergencies can arise from a wide range of causes, one of which includes outbreaks of contagion. The world has continued to be threatened by various infectious outbreaks of different types that have global consequences. An infectious disease doesn’t respect borders and can easily spread from a localized threat to one that puts at risk entire populations across the globe. Credible information, a resilient health system, and contingency planning can stop an epidemic and prevent it from turning into a pandemic^1^ an epidemic occurring over a very wide area, crossing international boundaries, and usually affecting a large number of people. Pandemics are identified by their geographic scale and reach (and when most people do not have immunity), rather than the severity of illness.^2^ Over the last 50 years, different parts of the world have seen at least 10 different disease outbreaks, from Marburg in 1967 to Ebola in 1976 to Middle East respiratory syndrome in 2012 and some, such as Ebola, resurfacing multiple times.^3^ However, with increasing infectious disease outbreaks frequency and intensity, there will be a continued need for global health security (GHS) resources.

## OVERVIEW OF THE GHS ALLIANCE

International collaboration, such as initiatives to improve the capacities of countries by strengthening health systems to protect people around the world against infectious diseases, might be a vital step to promote GHS and be better prepared to respond effectively to a global health crisis. The International Health Regulations (IHR) is a legally binding agreement for the coordination of the management of events that may constitute a public health emergency of international concern. Since 2005, the IHR has committed World Health Organization (WHO) member states to participate in international surveillance networks by reviewing and implementing sound surveillance strategies that contribute to global outbreak intelligence.^4^

However, in many ways, the frequent epidemics, such as severe acute respiratory syndrome (SARS) coronavirus, H1N1 influenza, and Ebola virus disease, during the past few years, have had devastating impacts at the country, regional, and global levels and were a wake-up call that exposed major weaknesses in the global system for addressing epidemic threats. In 2014, the worst outbreak of Ebola disease in West Africa the world has ever known unraveled. Nosocomial transmission, disruption of the health care system, and community upheaval are well-known features of this outbreak. The consequences were grave with over 30 000 Ebola cases, including approximately 11 000 dead from the disease, another 10 000 estimated deaths due to other health conditions going untreated, and millions of dollars lost in duplication of efforts due to poor coordination and communication across the global system.^5^

Nonetheless, this tragic event also brought forth a commitment to a new collective paradigm focus around GHS. The GHS agenda was launched in 2014 in collaboration with nations from around the world. While the goals of the post-Ebola GHS agenda are to accelerate progress toward a world safe and secure from infectious disease threats; promote GHS as an international priority; and spur progress toward full implementation of the WHO IHR 2005, the World Organization for Animal Health Performance of Veterinary Services pathway, and other relevant GHS frameworks through multilateral and multisectoral collaboration and sharing of best practices between partners (governmental, private, and non-governmental), global health threats posed by infectious diseases continue to date with increased frequency and intensity.^6^

## COVID-19 AND RESPONSE STRATEGIES

A pandemic has been under way since March 11, 2019. While all pandemics are unique in their level of transmission and breadth of impact, COVID-19 pandemic is the deepest global crisis of the 21st century, which has affected nearly every country globally. Globally, over 18.6 million confirmed cases, including over 700 000 deaths, were reported to the WHO as of August 6, 2020, from over 200 countries. COVID-19 cases have been reported across the African continent, with the number of confirmed cases passing 848 000 on August 6, 2020, and in Ethiopia with over 19 800 reported cases and 340 deaths as of the aforementioned date.^7^ Further impacts on the food security, employment, health care, and education have already been felt. People are missing work, as they are asked to self-quarantine and self-monitor, an entire school is closed, and similar other massive number of services are canceled.

Some authors suggest that the rapid spreading of COVID-19 and poorer outcomes for patients are potentially associated with health care resource availability.^8^ The lack of multidisciplinary teams to coordinate sectoral contribution and generation of evidence to improve programming, among others, also affects a quick and successful response.^9^ As the access to vaccines and antiviral drugs during a pandemic will be extremely limited, especially in countries with limited resources, nonmedical approaches such as social and behavior change measures may be the only way to delay the spread of the disease. A risk communication is a strategic effort that involves understanding and respecting local cultures, as well as multichannel integration, participation of relevant stakeholders, including communities and at-risk populations, and increased attention to evaluation.^10^

In this fight against COVID-19, it is important to ensure that relevant, accurate, and timely information is delivered to the public to inform, raise awareness, respond effectively during an outbreak, and teach the best practices for the community to take action to reduce their exposures and risks. The lack of accurate information on health issues and in a timely manner creates unacceptable room for rumors about how the new coronavirus disease is transmitted and treated, how to prevent infection, and how what constitutes safe or unsafe behavior might cause stigma and panic and fuel an epidemic.^11^ Individuals may be stigmatized if it becomes known that they have an illness of public health concern. Stigmatization may result from scientifically valid concerns or from unrealistic ones. For instance, much of the stigmatization of HIV/AIDS patients and people exposed to Ebola virus is derived from false beliefs about transmission.

It is already evident that when health systems are overwhelmed and people fail to access needed services, both direct mortality and indirect mortality from preventable and treatable conditions increase. The 2014 Ebola epidemic in West Africa indirectly caused more than 10 000 deaths due to HIV/AIDS, tuberculosis, and malaria.^5^ An understanding of this issue calls for increased attention to maintain essential health services in the pandemic context. The WHO has been providing critical support through guidelines, convening, and coordinated programs.^12,13^

As COVID-19 continues to impact local and global communities, state actors will need to make difficult choices to ensure that COVID-19 and other urgent, ongoing public health problems are addressed while minimizing risks to health workers and communities. The collection, analysis, and dissemination of accurate health information is critical for making effective policy and programmatic decisions that will ensure that resources get to where they are needed most. This requires democracy and transparency, as well as committed leadership that can communicate regularly and proactively and act in a way that conveys confidence, warmth, and strength. This, in turn, can be an important means of demonstrating respect, reducing stigma, promoting trust, and generating individual and community support for the pandemic response activities.^14^

## AFRICAN COUNTRIES’ APPROACHES

As noted in a commentary by Yan and Fujie,^15^ the COVID-19 outbreak reported on December 31, 2019, could have been contained, but limited capacity of early detection and response at its origin turned it into an epidemic of global proportions. Some commentators^16,17^ suggest that African countries still have the opportunity of early detection and response, by taking exceptional public health measures, as they have somewhere else, involving a lockdown of the city and mass isolation and/or quarantine, school closures, social distancing mandates, intense case finding and contact tracing, and testing and/or treatment before they are faced with widespread community transmission. They argue that, in many of these nations where health system weakness remains acute, inadequate surveillance, laboratory capacity for diagnostic tests, staff and financial resources, as well as uncontrolled population movement would facilitate the easy spread of the virus.

African countries have made a remarkable effort to coordinate among themselves toward the regional fight against COVID-19. This should be no surprise as solidarity is inherent to the social value of African Ubuntu, a form of leadership that is loosely translated as the essence of being human. With Ubuntu, a person’s humanity is bound with others. With Ubuntu, damage to 1 damages the whole. They established early the Africa Task Force for Novel Coronavirus in partnership with the African Union, Africa Centres for Disease Control and Prevention (CDC), and the WHO following an emergency ministerial meeting in Addis Ababa on February 22, 2020.^16^

The Africa CDC provides technical assistance to member states to improve their health systems. It is providing strong coordination and COVID-19 guidance, as well as strengthened national public health institutes, referral laboratories, laboratory and epidemiology workforces, infection control, and community networks are ready to be harnessed.^18^ An example of results of this effort has been the extension of laboratory capacity, from (in January) just 2 qualified laboratories in sub-Saharan Africa, to whereby laboratories in 46 African countries are now able to do COVID-19 testing.^18^

Testing strategies are key to ameliorating economic and social hardship, concentrating resources, and allowing more targeted interventions. However, the continent falls very short from the tens of millions of tests the United Nations estimates will be required – at least 74 million.^19^ There are several factors slowing testing; however, the need for diagnostics has never been higher.^20^ African governments have been working to procure these and other medical resources using national and donor funds. One of the biggest deliveries has been tens of thousands of diagnostic tests, masks, and other personal protective equipment to each African Union member state from the PM Abiy, Jack Ma and Alibaba Foundation Initiative to Reverse COVID-19 from Africa, but additional components need to be procured.^21^ The operation of the logistics and transportation to each state of this major medical equipment donation provided by the aforementioned initiative has primarily been coordinated by the Ethiopian Prime Minister, Dr Abiy Ahmed.

While the rapid reaction appears to have slowed the impact of COVID-19 on a continent, a highly transmissible and fast spreading virus coupled with the vulnerability and fragile health systems can quickly overwhelm important capacities created by recent investments. This demonstrates therefore that domestic funds must be mobilized, while bilateral and multilateral aid must also be increased to prepare for the worst. Home-based care combined with widespread testing and early diagnosis has been shown elsewhere, resulting in a lower mortality rate.^20,22^ Given almost non-existent critical care beds in the region, Matthew and colleagues suggest that such home-based care could be a model for African contexts, building on community-level response experiences from the West African Ebola outbreak.^20^

Yet, approaches and methods may vary across different countries or different locations of a given country, according to their risk levels, people’s perceptions, needs, local capacities, and current situations, and a wide range of public health and social measures has been taken and will continue in the short and longer term. Generally, these measures comprise increasing or decreasing limits on human contact, travel, and business operations. Ethiopia, the second populous nation in Africa with over 100 million people, declared a state of emergency, postponed highly anticipated elections, and closed borders, but has not implemented a lockdown of movement in the country.

## ETHICS IN PANDEMICS

Pandemic response policies and processes all need to be trusted for an effective and ethical pandemic response. There exist diverse ethical questions that may be raised during pandemics response, of which relate, first, to measures that required to protect the greater good of society including, surveillance, mandatory testing and/or treatment, and isolation and/or quarantine. Each of these measures could interfere with basic human rights and liberties, and with autonomy, in particular.^23^ In the existing public health ethics framework,^24^ the use of coercive measures might be justified in the context of an epidemic or other emergency if it is necessary to prevent the greatest harm. Plans for such measures should also ensure safe, habitable, and humane conditions of confinement, including the provision of basic necessities.^25^

A second issue relates to practices such as those noted above, often posing limited apparent physical harm and may go against the cultural, moral, or religious beliefs of individuals and communities.^26^ The relative weight given to each harm and benefit depends, however, on the context and on moral and cultural beliefs. “For example, closing schools during a pandemic might appear to some communities a reasonable way to protect children from spreading an illness; in other communities, however, schools may be the safest, most nurturing settings for children, and, without access to them, children may be put at risk of more serious harm.”^27^ These conflicting perspectives, in turn, indicate the importance of involving communities that are affected by emergencies in making decisions about potential harm and benefits.

Third, when responses to emergencies involve novel medical resources, issues of equity or access are quick to emerge. While the principle of equitable distribution of resources to coordinate emergency responses is ethically compelling, the existence of power imbalances and commercial pressures continues to make its application highly contested. There were situations that reveal the global dimension of unequal access to scarce diagnostics and life-saving treatments. Resources that might be limited during a public health emergency include vaccines; medications to treat infected persons; life-sustaining medical technologies, such as ventilators; hospital beds, particularly in intensive care; laboratory capacity for diagnostic tests; access to medical specialists; and infection control equipment. Fights for access to antiretroviral therapy in the 1980s are well-known examples.^20^ Currently, this situation can be avoided. Ethically relevant criteria for global allocation of scarce resources are a critical part in the global effort for pandemic response and GHS. Hence, the global community should continue the efforts to create mechanisms for the equitable distribution of scarce resources.

Ultimately, research is poised to become an integral part of the response to emergencies with public health consequences. However, ethical questions such as “research for whom?” and “research to what end?” are often raised but remain little discussed. While studies of how individuals and organizations react in the short term to the actual onset of epidemics and other emergencies remain common, studies of longer-term adjustments are rare, such as mitigation and the physical and social changes made during periods of normalcy with the intent of reducing losses in future global health threats. Therefore, a grassroots strategy that would empower those, especially vulnerable, to improve their overall situation continues to be sought.

## CONCLUDING REMARKS

COVID-19 has revealed how the world is strikingly unprepared for a pandemic and how viruses easily spread in this interconnected world. Yet, going forward, with the infectious disease outbreaks becoming more common, it can be hoped that the GHS resources – surveillance, laboratory, workforce, and more – will be strengthened. This is critical to both keeping pace with diseases that spread with the severity and rapidity of COVID-19 and being more resilient in the face of future global health threats. As the world can learn during the past few years about frequent infectious disease outbreaks, (these) diseases respect no borders, and, therefore, our spirit of solidarity must respect no borders in our efforts to stop the ongoing COVID-19 pandemic and be better prepared to respond effectively to a health crisis in the future. Given the increasing rate of growth and interconnectedness of the global population and the resulting consumption and infringement on the environment, it can be predicted that zoonotic diseases and outbreaks will continue to surface. The dynamics of pandemic outbreaks require effective engagement, coordination, and cooperation among a wide range of sectors and actors with the goal of optimal health for the people, animals, and environment.
